# Supplementary material for: Development and Validation of a One-Step Reverse Transcription Real-Time PCR Assay for Simultaneous Detection and Identification of Tomato Mottle Mosaic Virus and Tomato Brown Rugose Fruit Virus
Source: Plants (Basel). 2022 Feb 11;11(4):489. doi: 10.3390/plants11040489 (PMC8878898; doi:10.3390/plants11040489)
Supplement: Supplementary file 1 [file plants-11-00489-s001.zip › plants-1556882 -Supplementary.pdf]

Supplementary Table S1. Isolates and material used in this study

Virus	ID	Collection	Origin	plant species/cv.	sampling	Matrix
BPeMV	BN-4708	DSMZ	Netherlands	*S. melongena*	before 1988	leaf***
CGMMV	PV-0375	DSMZ	Germany	*Cucumis sp.*	Before 1992	leaf
CGMMV	NIB V271	at NIB maintained since 2012 (origin: NVWA, isolate 4073020-A)	Netherlands	*Cucumis sativus*	nd	leaf
CGMMV	NIB V320	at NIB maintained since 2018 (origin: Murcia. Miguel Aranda)	Spain	*Cucumis sativus*	nd	leaf
Healthy	MR 5	CREADC	Italy	*S.lycopersicum*		leaf
Healthy	SanoPep	CREADC	Italy	*C.annuum*		leaf
Healthy	MR5-s	CREADC	Italy	*S.lycopersicum*		seeds
Healthy	SanoPep-s	CREADC	Italy	*C.annuum*		seeds
ObPV	PV-1176	DSMZ	Hungary	*C.annuum*	before 2015	leaf***
ORSV	PV-1048	DSMZ	Germany	Orchis spp.	before 2007	leaf***
PaMMV	PV-0606	DSMZ	Greece	*C.annuum*	before 2000	leaf***
PMMoV	PV-0165	DSMZ	nd	*C.annuum*	before 2019	leaf***
RMV	PV-0145	DSMZ	nd	*Plantago media*	before 1988	leaf***
SFBV	PV-1058	DSMZ	nd	*Streptocarpus spp.*	before 2012	leaf***
SHMV	PV-0156	DSMZ	nd	*Phaseolus vulgaris*	before 1988	leaf***
TMGMV	PV-0124*	DSMZ	Italy	nd	before 1988	leaf***
TMV	NIB V037	NIB	Slovenia	*S.lycopersicum*	2000	leaf
TMV	PV-0137	DSMZ	Germany	nd	before 1988	leaf***
TMV	PV-1252	DSMZ	nd	*Nicotian tabacum*	2019	leaf***
ToBRFV	MR50-Tob-SIC22/19-T	CREADC	Italy	*S.lycopersicum*		leaf
ToBRFV	MR50-Tob-SIC22/19-T-s	CREADC	Italy	*S.lycopersicum*		seeds
ToBRFV	Tob-SIC22/19-P	CREADC	Italy	*C.annuum*		leaf
ToBRFV	Tob-SIC25/19-T	CREADC	Italy	S.lycopersicum		leaf
ToBRFV	Tob-Pie105/19-T	CREADC	Italy	S.lycopersicum		leaf
ToBRFV	Tob-Pie105/19-P	CREADC	Italy	*C.annuum*		leaf
ToBRFV	MR50 -10-5**	CREADC	Italy	*S.lycopersicum*		leaf
ToBRFV	S21	Volcani center	Israel	*S.lycopersicum*	nd	leaf
ToBRFV	S22	Volcani center	Israel	*S.lycopersicum*	nd	leaf
ToMMV	S1	IBMCP	Spain	*Nicotiana benthamiana*	2019	leaf
ToMMV	S2	IBMCP	Spain	*Nicotiana benthamiana*	2019	leaf
ToMMV	PV-1267*	DSMZ	California (USA)	*S.lycopersicum*	2016	leaf***
ToBRFV/ToMMV§	Pep-271	CREA-DC	Italy -Battipaglia	*C. annuum*/'Almuden'	2021	leaf
ToBRFV/ToMMV§	Pep-282	CREA-DC	Italy- Fondi	*C. annuum*/'Achille'	2021	leaf
ToBRFV/ToMMV§	Pep-284	CREA-DC	Italy- Fondi	*C. annuum*/'Luvanor'	2021	leaf
ToBRFV/ToMMV§	Pep-288	CREA-DC	Italy- Fondi	*C. annuum*/'Coraza'	2021	leaf
ToBRFV/ToMMV§	Papr-327	CREA-DC	Italy-Termoli	*C. annuum*/'Cornetto'	2021	leaf
ToBRFV/ToMMV§	Pep-350	CREA-DC	Italy-Foggia	*C. annuum*/'Ricardo'	2021	leaf
ToBRFV/ToMMV§	Pom-25	CREA-DC	Italy-Rome	*S.lycopersicum*/'Roma'	2021	leaf
ToBRFV/ToMMV§	Pom-222	CREA-DC	Italy- Battipaglia	*S.lycopersicum*/'Dobles'	2021	leaf
ToBRFV/ToMMV§	Pom-241-Fo	CREA-DC	Italy- Battipaglia	*S.lycopersicum*/'sv5197'	2021	leaf
ToBRFV/ToMMV§	Pom-241-Fr	CREA-DC	Italy- Battipaglia	*S.lycopersicum*/'sv5197'	2021	fruit
ToBRFV/ToMMV§	Pom-311	CREA-DC	Italy-Termoli	*S.lycopersicum*/'Impact'	2021	leaf

ToBRFV/ToMMV§	Pom-361	CREA-DC	Italy-Foggia	*S.lycopersicum*/'Pretender'	2021	leaf
ToMV	NIB V036	NIB	Slovenia	*Petunia*	2001	leaf
ToMV	NIB V049	NIB	Slovenia	nd	nd	leaf
ToMV	NIB V072	NIB	Slovenia	*S.lycopersicum*	2004	leaf
ToMV	NIB V104	NIB	Slovenia	*S.lycopersicum*	2006	leaf
ToMV	PV-0141	DSMZ	France	*C.annuum*	before 1988	leaf***
YMoV	PV-0527	DSMZ	Germany	*Impatiens spp.*	before 1988	leaf***

nd-not determined *purchased independently by CREA and NIB
**100.000 dilution
***dry-lyophilized leaf material
§ToBRFV/ToMMV mixed infected samples

Supplementary Table S2. RNA samples previously tested for ToBRFV, and further tested with the duplex assay developed in this study.

							Singleplex (M&W)	Duplex	Duplex	
ID	Plant species	Cultivar	Matrix	Origin	Date	ToBRFV status	ToBRFV	ToBFRV	ToMMV	virus detected by sanger sequencing of nested PCR product of Dovas et al. (2004)
4	*C.annuum*	nd	seeds	Italy	03/2021	negative	NA	NA	NA	nt
5	*C.annuum*	nd	seeds	Italy	03/2021	negative	NA	NA	NA	nt
6	*C.annuum*	nd	seeds	Italy	03/2021	negative	NA	NA	NA	nt
7	*C.annuum*	nd	seeds	Italy	03/2021	negative	NA	NA	NA	nt
8	*C.annuum*	nd	seeds	Italy	03/2021	negative	NA	NA	NA	nt
9	*C.annuum*	nd	seeds	Italy	03/2021	negative	NA	NA	NA	nt
10	*C.annuum*	nd	seeds	Italy	03/2021	negative	NA	NA	NA	nt
20	*C.annuum*	nd	seeds	Italy	03/2021	negative	NA	NA	NA	nt
21	*C.annuum*	nd	seeds	Italy	03/2021	negative	NA	NA	NA	nt
22	*C.annuum*	nd	seeds	Italy	03/2021	negative	NA	NA	NA	nt
23	*C.annuum*	nd	seeds	Italy	03/2021	negative	NA	NA	NA	nt
33	*S. lycopersicum*	nd	seeds	Italy	05/2021	negative	NA	NA	NA	nt
34	*S. lycopersicum*	nd	seeds	Italy	05/2021	negative	NA	NA	NA	nt
35	*S. lycopersicum*	nd	seeds	Italy	05/2021	negative	NA	NA	NA	nt
36	*S. lycopersicum*	nd	seeds	Italy	05/2021	negative	NA	NA	NA	nt
37	*S. lycopersicum*	nd	seeds	Italy	05/2021	negative	NA	NA	NA	nt
38	*S. lycopersicum*	nd	seeds	Italy	05/2021	negative	NA	NA	NA	nt
39	*S. lycopersicum*	nd	seeds	Italy	05/2021	negative	NA	NA	NA	nt
40	*S. lycopersicum*	nd	seeds	Italy	05/2021	negative	NA	NA	NA	nt
50	*S. lycopersicum*	nd	seeds	Italy	05/2021	negative	NA	NA	NA	nt
51	*S. lycopersicum*	nd	seeds	Italy	05/2021	negative	NA	NA	NA	nt
52	*S. lycopersicum*	nd	seeds	Italy	05/2021	negative	NA	NA	NA	nt
53	*S. lycopersicum*	nd	seeds	Italy	07/2021	negative	NA	NA	NA	nt
54	*S. lycopersicum*	nd	seeds	Italy	07/2021	negative	NA	NA	NA	nt
55	*S. lycopersicum*	nd	seeds	Italy	07/2021	negative	NA	NA	NA	nt
56	*S. lycopersicum*	nd	seeds	Italy	07/2021	negative	NA	NA	NA	nt
60	*C.annuum*	nd	seeds	Italy	07/2021	negative	NA	NA	NA	nt
61	*C.annuum*	nd	seeds	Italy	07/2021	negative	NA	NA	NA	nt
62	*C.annuum*	nd	seeds	Italy	07/2021	negative	NA	NA	NA	nt
63	*C.annuum*	nd	seeds	Italy	07/2021	negative	NA	NA	NA	nt
64	*S. lycopersicum*	nd	seeds	Italy	07/2021	negative	NA	NA	NA	nt
65	*S. lycopersicum*	nd	seeds	Italy	07/2021	negative	NA	NA	NA	nt
66	*S. lycopersicum*	nd	seeds	Italy	07/2021	negative	NA	NA	NA	nt
67	*S. lycopersicum*	nd	seeds	Italy	08/2021	negative	NA	NA	NA	nt
68	*C.annuum*	nd	seeds	Italy	08/2021	negative	NA	NA	NA	nt
69	*C.annuum*	nd	seeds	Italy	08/2021	negative	NA	NA	NA	nt
72	*C.annuum*	nd	seeds	Italy	08/2021	negative	NA	NA	NA	nt
73	*S. lycopersicum*	nd	seeds	Italy	08/2021	negative	NA	NA	NA	nt
74	*S. lycopersicum*	nd	seeds	Italy	08/2021	negative	NA	NA	NA	nt
82	*S. lycopersicum*	nd	seeds	Italy	08/2021	negative	NA	NA	NA	nt
83	*S. lycopersicum*	nd	seeds	Italy	08/2021	negative	NA	NA	NA	nt
84	*S. lycopersicum*	nd	seeds	Italy	08/2021	negative	NA	NA	NA	nt
90	*C.annuum*	nd	seeds	Italy	08/2021	negative	NA	NA	NA	nt

91	*C.annuum*	nd	seeds	Italy	08/2021	negative	NA	NA	NA	nt
92	*C.annuum*	nd	seeds	Italy	08/2021	negative	NA	NA	NA	nt
D1/21	*S. lycopersicum*	Fuji Pink	seeds	Brazil	01/2021	negative	NA	NA	NA	nt
D2/21	*S. lycopersicum*	Sweet million	seeds	China	01/2021	negative	NA	NA	NA	nt
D3/21-A,B,C	*S. lycopersicum*	Val	seeds	Slovenia	01/2021	negative	NA*	NA	NA	nt
D4/21	*C.annuum*	Belladonna	seeds	India	01/2021	negative	NA	NA	NA	nt
D28/21	*S. lycopersicum*	Dyno F1	seeds	Thailand	02/2021	negative	NA	NA	NA	nt
D41/21	*S. lycopersicum*	Runner F1	seeds	nd	03/2021	negative	NA	NA*	NA	nt
D43/21-A,B,C	*S. lycopersicum*	Begunec	seeds	Slovenia	03/2021	negative	NA*	NA*	NA	nt
D76/21-A,B,C	*C.annuum*	Alpina	seeds	Serbia	03/2021	negative	NA	NA	NA	nt
D191/21-A,B,C	*S. lycopersicum*	Sweetheart	seeds	China	04/2021	negative	NA	NA	NA	nt
D194/21-A,B,C	*C.annuum*	Karola	seeds	China	04/2021	negative	38.2**	NA	NA	nt
D195/21-A,B,C	*C.annuum*	Stef	seeds	China	04/2021	negative	NA*	NA	NA	nt
D196/21-A,B,C	*C.annuum*	Dumbo	seeds	China	04/2021	negative	NA	NA	NA	nt
D198/21-A,B,C	*C.annuum*	Dracula	seeds	China	04/2021	negative	35.8	NA*	NA	nt
D261/21-A,B,C	*S. lycopersicum*	Coure di bue	seeds	China	04/2021	negative	NA	NA	NA*	nt
D262/21-A,B	*C.annuum*	Cayenna	seeds	China	04/2021	negative	NA	NA	NA	nt
D263/21-A,B,C	*S. lycopersicum*	Pantano	seeds	China	04/2021	negative	NA	NA*	NA*	nt
D264/21-A,B,C	*C.annuum*	Corno giallo	seeds	China	04/2021	negative	NA	NA	NA*	nt
D278/21-A,B,C	*S. lycopersicum*	Marmande	seeds	China	04/2021	negative	37.1**	36.4**	NA	nt
D279/21-A,B	*C.annuum*	Quadrato d'Asti giallo	seeds	China	04/2021	negative	NA	NA*	NA	nt
D280/21-A,B	*C.annuum*	Soroksari	seeds	China	04/2021	negative	NA	NA	NA	nt
D281/21-A,B,C	*S. lycopersicum*	Red cherry	seeds	China	04/2021	negative	NA	NA	NA*	nt
D308/21-A,B,C	*S. lycopersicum*	Ghittia	seeds	China	05/2021	negative	NA	NA	NA*	nt
D311/21-A,B,C	*S. lycopersicum*	Lillagro	seeds	China	05/2021	negative	NA	NA	NA*	nt
D313/21-A,B,C	*S. lycopersicum*	Corina	seeds	China	05/2021	negative	38.3**	38.7**	NA	nt
D316/21-A,B,C	*S. lycopersicum*	Henriet	seeds	China	05/2021	negative	NA*	NA*	NA*	nt
D317/21-A,B,C	*C.annuum*	Andreika	seeds	China	05/2021	negative	NA	NA	NA*	nt
D318/21-A,B,C	*C.annuum*	Barbara	seeds	China	05/2021	negative	NA	NA	NA	nt
D319/21-A,B,C	*C.annuum*	Splendid	seeds	China	05/2021	negative	NA*	NA	NA	nt
D320/21-A,B,C	*C.annuum*	Vlad	seeds	China	05/2021	negative	NA*	NA	NA	nt
D321/21-A,B,C	*C.annuum*	Mircea	seeds	China	05/2021	negative	NA	NA	NA	nt
D322/21-A,B,C	*C.annuum*	California Wonder	seeds	China	05/2021	negative	NA	NA*	NA*	nt
D325/21-A,B,C	*S. lycopersicum*	Raluca	seeds	China	05/2021	negative	NA*	NA*	NA*	nt
D327/21-A,B,C	*S. lycopersicum*	Lillagro	seeds	China	05/2021	negative	37.7**	NA*	NA*	nt
D329/21-A,B,C	*C.annuum*	Karola	seeds	China	05/2021	negative	34.8	NA*	NA	nt
D335/21-A,B,C	*C.annuum*	Vlad	seeds	China	05/2021	negative	36	NA	NA	nt
D979/21-A,B,C	*S. lycopersicum*	Factor F1	seeds	nd	08/2021	negative	NA*	NA	NA	nt
D1364/21	*S. lycopersicum*	Toivo F1	seeds	nd	10/2021	negative	NA	NA	NA	nt
D1388/21	*S. lycopersicum*	Vitellio	seeds	nd	10/2021	negative	NA	NA	NA	nt
D1365/21-A,B,C	*S. lycopersicum*	Tonatico	seeds	nd	10/2021	negative	NA*	NA	37.7**	no nested PCR product obtained
D324/21-A,B,C	*S. lycopersicum*	Elisabeta	seeds	China	05/2021	negative	NA*	NA*	37.0**	ToMV confirmed
D201/21-A,B,C	*S. lycopersicum*	Ghittia	seeds	China	04/2021	negative	NA*	NA*	36.8	no nested PCR product obtained
D310/21-A,B,C	*S. lycopersicum*	Ideal	seeds	China	05/2021	negative	NA*	NA*	36.6**	ToMV confirmed

D202/21-A,B,C	*S. lycopersicum*	Drops	seeds	China	04/2021	negative	NA*	NA*	36.2**	ToMV suspected
D309/21-A,B,C	*S. lycopersicum*	Unibac	seeds	China	05/2021	negative	NA	NA	36.0**	ToMV confirmed
D323/21-A,B,C	*S. lycopersicum*	Amalia	seeds	China	05/2021	negative	NA*	NA*	35.8	TMGMV suspected
D333/21-A,B,C	*S. lycopersicum*	Elisabeta	seeds	China	05/2021	negative	NA	NA*	35.6	mix infection with different tobamoviruses suspected
D326/21-A,B,C	*S. lycopersicum*	Buzau	seeds	China	05/2021	negative	37.3**	37.9**	31.7	mix infection with different tobamoviruses suspected
D200/21-A,B,C	*S. lycopersicum*	Raluca	seeds	China	04/2021	negative	36.9	35.2	29.7	mix infection with ToMMV and ToMV suspected
D328/21-A,B,C	*S. lycopersicum*	Chiquita pot	seeds	China	05/2021	negative	37.8	36.6**	29	ToMMV confirmed
D334/21-A,B,C	*S. lycopersicum*	Ruxandra	seeds	China	05/2021	negative	38.5**	NA	27.7	ToMMV confirmed
D314/21-A,B,C	*S. lycopersicum*	Sandybelle	seeds	China	05/2021	negative	NA*	NA*	27.6	ToMMV confirmed
D315/21-A,B,C	*S. lycopersicum*	Imola	seeds	China	05/2021	negative	NA*	NA*	26.2	ToMMV confirmed
D332/21-A,B,C	*S. lycopersicum*	Amalia	seeds	China	05/2021	negative	36.4**	36.0**	23.1	ToMMV confirmed
D192/21-A,B,C	*S. lycopersicum*	Silvia	seeds	China	04/2021	positive	31.3	31.9	NA	nt
D193/21-3	*S. lycopersicum*	Drops	seeds	China	04/2021	positive	32.6	31.6	NA	nt
D197/21-A,B,C	*C.annuum*	Barbara	seeds	China	04/2021	positive	29.2	29	NA	nt
D199/21-A,B,C	*C.annuum*	Pintea	seeds	China	04/2021	positive	17.2	16.1	NA	nt
D312/21-1	*S. lycopersicum*	Chiquita pot	seeds	China	05/2021	positive	32.1	31.7	NA	nt
D330/21-A,B,C	*C.annuum*	Galben superior	seeds	China	05/2021	positive	32.7	33.8	NA	nt
D330/21-2,4	*C.annuum*	Galben superior	seeds	China	05/2021	positive	31.7	32.5	NA*	nt
D331/21-4	*C.annuum*	Stef	seeds	China	05/2021	positive	31.9	33	NA*	nt

Each sample was divided into three subsamples, each of which was tested in 2-3 tehnical repetitions. The average Cq values of all parallels are indicated or marked as NA if no exponential amplification curves were observed.

ToBRFV status was determined based on results of both real-time RT-PCRs recomended in EPPO standard PM7/146(1)

nd - not determined; nt - not tested; *one up to half parallels with Cq between 34 and 40; **one up to half parallels with no exponential amplification curve

Supplementary Table S3. List of all the ToMMV isolates included in the in-silico analysis

**Isolate**	**Host**	**Origin**	**Sequencing methodology**	**Date of collection**	**Reference**
MN654021_ToMMV_19_02305	*C. annuum*	NL	Sanger HTS Illumina	2020	Australas. Plant Dis. Notes 15 (1), 8 (2020)
MH128145_ToMMV_CpB1	*S.lycopersicum*	BR	Illumina	1992	Nagai,A. et al., 2018
MG171192_MP_ToMMV_Hainan	*S.lycopersicum*	CN	Sanger	2016	Zhan,B. et al., 2017
KX898034_ToMMV_CA16_01	*S.lycopersicum*	USA	Sanger Illumina	2016	Sui,X. Et al., 2017
KX898033_ToMMV_SC13_05	*S.lycopersicum*	USA	Sanger Illumina	2013	Sui,X. Et al., 2018
KR824951_ToMMV_TiLhaLJ	*C. frutescens*	Tibet	nd	2013	Li et al., 2016
KR824950_ToMMV_YYMLJ	*C. annuum*	CN Yunnan	nd	2013	Li et al., 2017
KT810183_ToMMV_NY_13	*S.lycopersicum*	USA-NY	Illumina	2013	Padmanabhan,C. et al., 2015
KP202857_ToMMV_10_100	*S.lycopersicum*	USA-FL	Sanger IonTorrent	2010	Fillmer,K et al., 2015
MW582804_ToMMV DSMZ_PV_1267	*S.lycopersicum*	USA-california	Illumina	nd	Knierim,D et al., 2021
MN853592_ToMMV_LN	*S.lycopersicum*	CN Liaoning	Sanger	2015	Tu and Ji 2019
MH381817_ToMMV_HN	*S.lycopersicum*	CN	nd	nd	Liu e Zhou 2018
NC_022230_MP_ToMMV_MX5	*S.lycopersicum*	MX	sanger Illumina	2009	Li et al., 2013
KU594507_ToMMV_SP	*S.lycopersicum*	SP	Sanger	2015	Ambros,S et al., 2017

nd-not determined
