# Supplementary figures and images for: Development and Validation of a One-Step Reverse Transcription Real-Time PCR Assay for Simultaneous Detection and Identification of Tomato Mottle Mosaic Virus and Tomato Brown Rugose Fruit Virus
Source: Plants (Basel). 2022 Feb 11;11(4):489. doi: 10.3390/plants11040489 (PMC8878898; doi:10.3390/plants11040489)

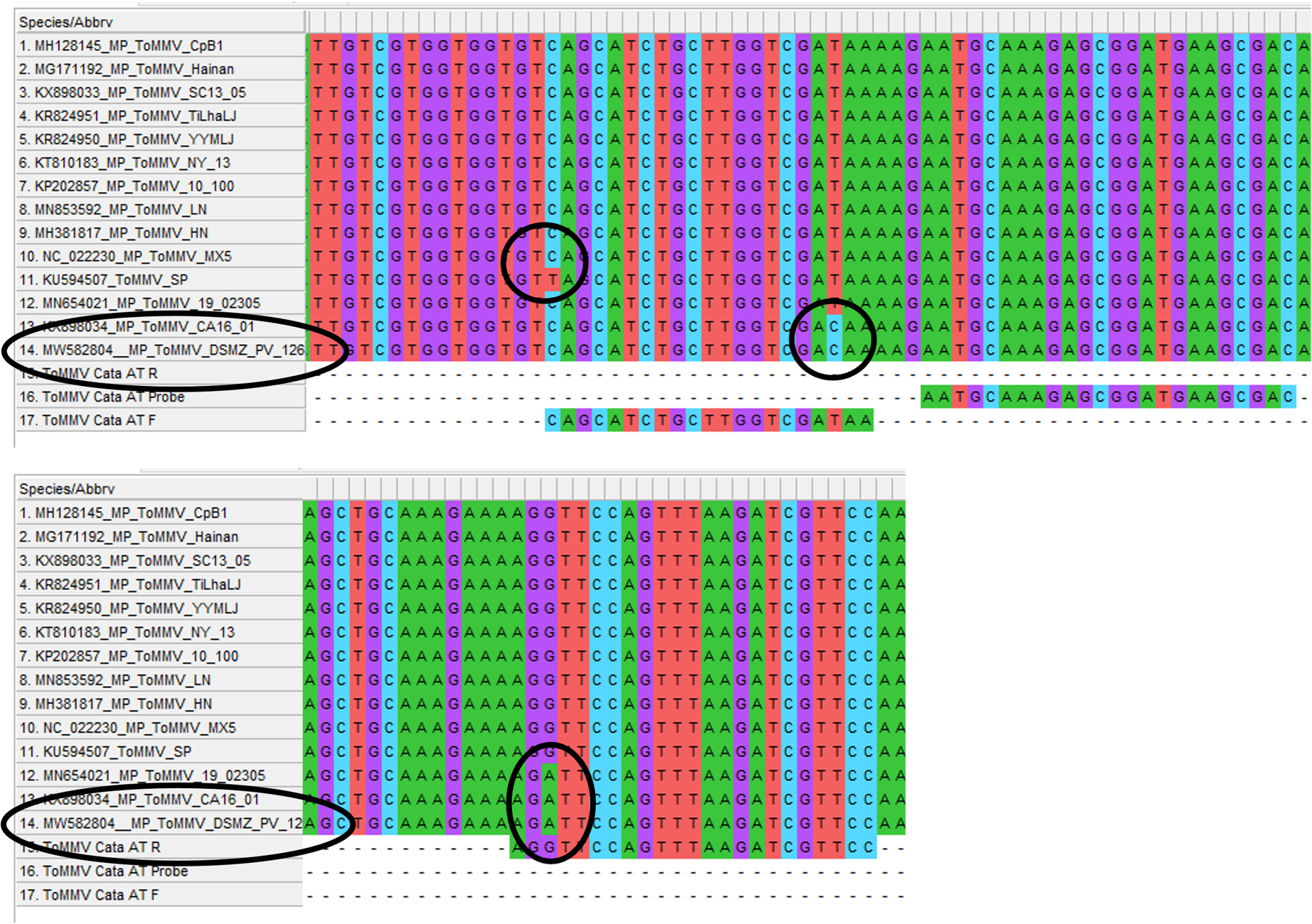

Supplement: Supplementary file 1 [file plants-11-00489-s001.zip › Supplementary Figure S1.tif]

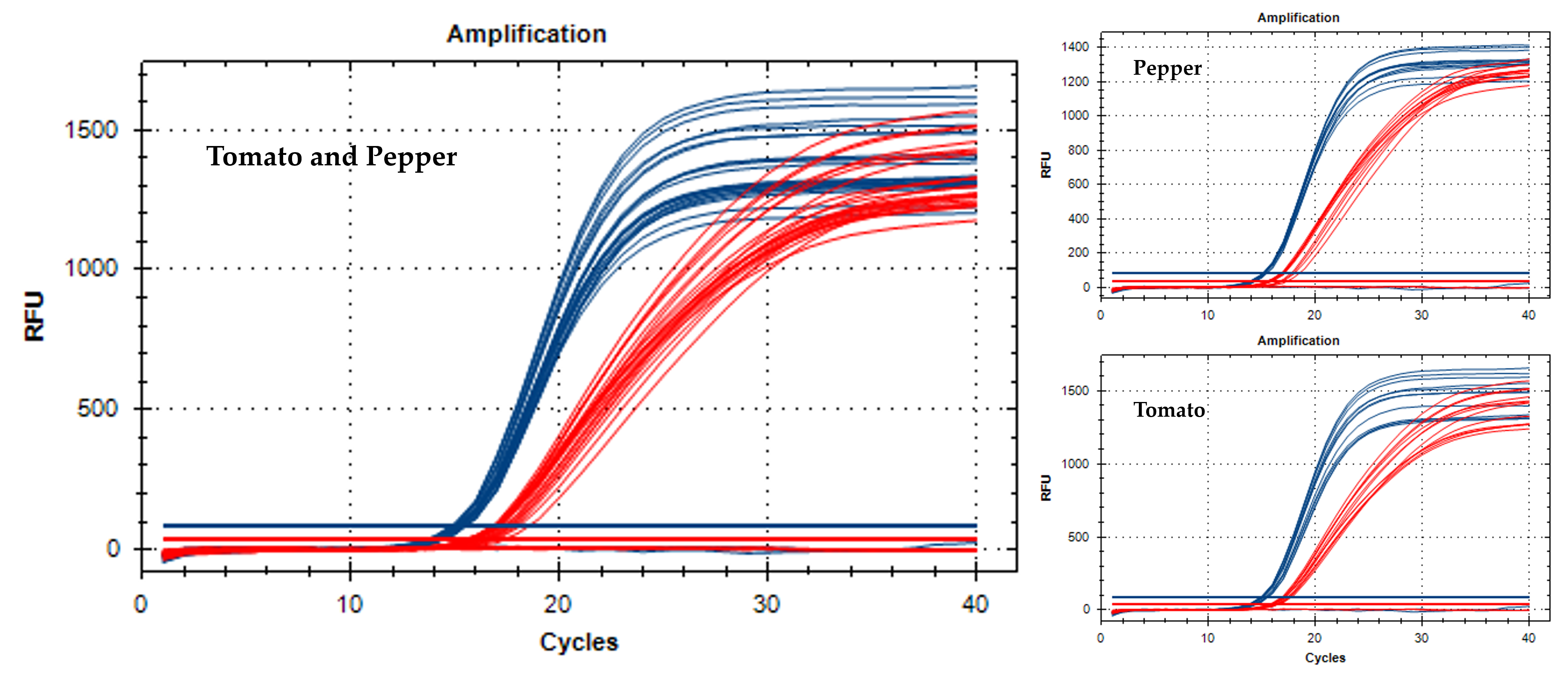

Supplement: Supplementary file 1 [file plants-11-00489-s001.zip › Supplementary Figure S2.tif]
